# Supplementary material for: Splice-Junction-Based Mapping of Alternative Isoforms in the Human Proteome
Source: Cell Rep. Author manuscript; Available in PMC 2020 Jan 15. (PMC6961840; doi:10.1016/j.celrep.2019.11.026)
Supplement: 2 [file NIHMS1546469-supplement-2.zip › DF1/PXD010154/Ovary/Ovary_21_AMHR2_RRGGVPTSHPPGAALPQTLMGLLPTAVEAPPNR.pdf]

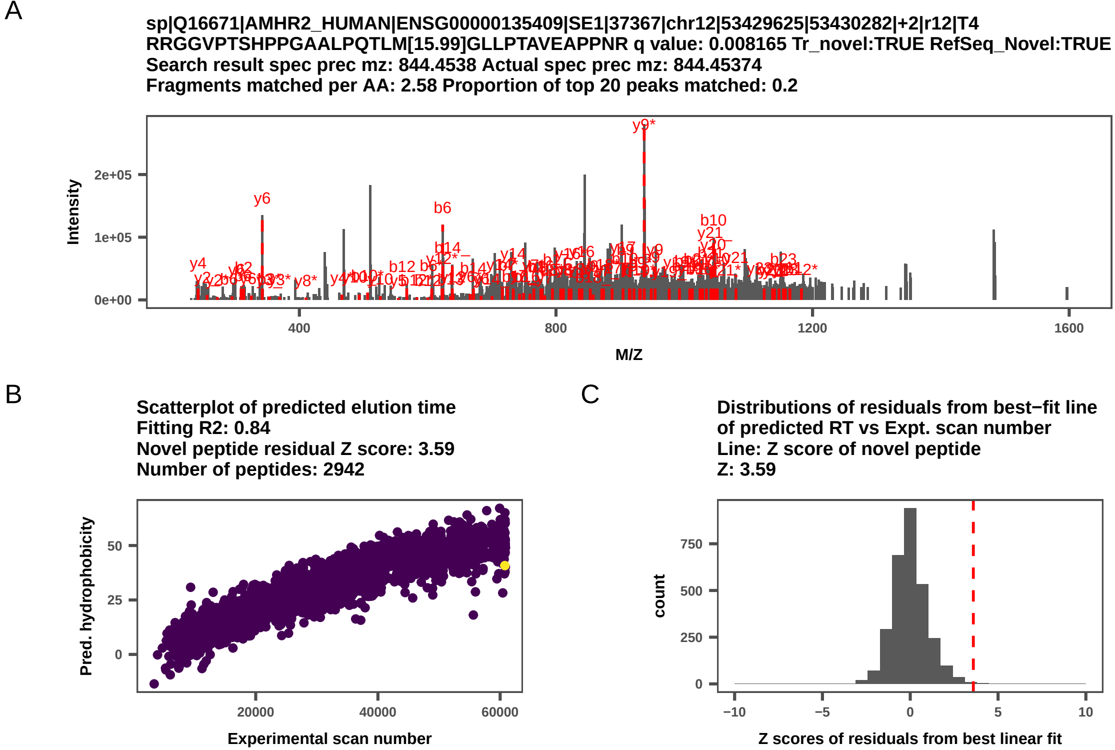
A
sp|Q16671|AMHR2_HUMAN|ENSG00000135409|SE1|37367|chr12|53429625|53430282|+2|r12|T4
RRGGVPTSHPPGAALPQTLM[15.99]GLLPTAVEAPPNR q value: 0.008165 Tr_novel:TRUE RefSeq_Novel:TRUE
Search result spec prec mz: 844.4538 Actual spec prec mz: 844.45374
Fragments matched per AA: 2.58 Proportion of top 20 peaks matched: 0.2
Intensity
2e+05
1e+05
0e+00
y9*
y6
b6
b10
y21
y4
400
800
1200
1600
M/Z
B
Scatterplot of predicted elution time
Fitting R2: 0.84
Novel peptide residual Z score: 3.59
Number of peptides: 2942
Pred. hydrophobicity
50
25
0
20000
40000
60000
Experimental scan number
C
Distributions of residuals from best−fit line
of predicted RT vs Expt. scan number
Line: Z score of novel peptide
Z: 3.59
count
750
500
250
0
−10
−5
0
5
10
Z scores of residuals from best linear fit
